# HisgAtlas 1.0: a human immunosuppression gene database

**DOI:** 10.1093/database/bax094

**Published:** 2017-12-15

**Authors:** Yuan Liu, Mengqi He, Dan Wang, Lihong Diao, Jinying Liu, Li Tang, Shuzhen Guo, Fuchu He, Dong Li

**Affiliations:** 1 State Key Laboratory of Proteomics, Beijing Proteome Research Center, National Center for Protein Sciences, Beijing Institute of Lifeomics, Beijing 102206, China; 2 School of Chinese Medicine, Beijing University of Chinese Medicine, Beijing 100029, China

## Abstract

Immunosuppression is body’s state in which the activation or efficacy of immune system is weakened. It is associated with a wide spectrum of human diseases. In the last two decades, tremendous efforts have been made to elucidate the mechanism of hundreds of immunosuppression genes. Immunosuppression genes could be valuable drug targets or biomarkers for the immunotherapeutic treatment of different diseases. However, the information of all previously identified immunosuppression genes is dispersed in thousands of publications. Here, we provide the HisgAtlas database that collects 995 previously identified human immunosuppression genes using text mining and manual curation. We believe HisgAtlas will be a valuable resource to search human immunosuppression genes as well as to investigate their functions in further research.

**Database URL**: http://biokb.ncpsb.org/HisgAtlas/

## Introduction

Immunosuppression is body’s state in which the activation or efficacy of immune system is weakened. Immunosuppression is associated with a wide spectrum of human diseases, such as autoimmune diseases, allergy, organ transplantation rejection and chronic infectious diseases (1). Most importantly, tumor can exploit immunosuppression mechanisms by co-opting certain immune checkpoint pathways to evade the immune system (2). Clinically, immunosuppression has been a promising therapy. For example, the immunosuppressive drugs like adalimumab (3) and abatacept (4) have been developed for the treatment of autoimmune disease. The ability of tolerogenic dendritic cells to induce and maintain immunotolerance has been exploited to resolve the side effects of non-specific inhibition of immune responses in organ transplantation (5). What’s more, immune checkpoint blockade therapy has made a significant effect in the treatment of cancer (2). Recently, FDA approves the first cancer treatment for any solid tumor with a specific genetic feature, which works by targeting the programmed cell death-1 (PD-1)/PD-L1 immune checkpoint pathway (6).

In the last two decades, tremendous efforts have been made to elucidate the molecular mechanism of human immunosuppression. Hundreds of immunosuppression genes (7) have been found to play vital roles in the induction, maintenance or destruction of immunosuppression (8). Some genes are associated with autoimmune diseases. Massive production of the immunoregulatory cytokine transforming growth factor–β by phagocytic cells can block self-perpetuating inflammation, which is the hallmark of all autoimmune responses (1). Autoimmune regulator gene, a histone-binding module, can promote self-tolerance and prevent organ-specific autoimmunity through the mediation of the thymic display of peripheral tissue antigens (9). Some gene products are valuable targets for the cancer immune checkpoint blockade therapy. For example, by inhibiting the proliferation of T cell, the PD-1 receptor can compromise anti-viral and antitumor T cell responses (10). Blockade of the PD-1/PD-L1 pathway can active antitumor immune responses and has been a very successful therapy for cancer (2).

Besides the known targets, alternative immune checkpoints like TIM3, LAG3 are also promising for the cancer immune checkpoint blockade therapy. Their upregulation has been found to be associated with adaptive resistance to therapeutic PD-1 blockade. As Hammerman *et al.* (11) said, ‘Responses to PD-1/PD-L1 therapy remain suboptimal in the majority of patients and there is much to learn and improve on,’ showing the importance of finding new promising immune checkpoints. In fact, their inhibitors have already entered clinical experiment (12).

**Figure 1.**
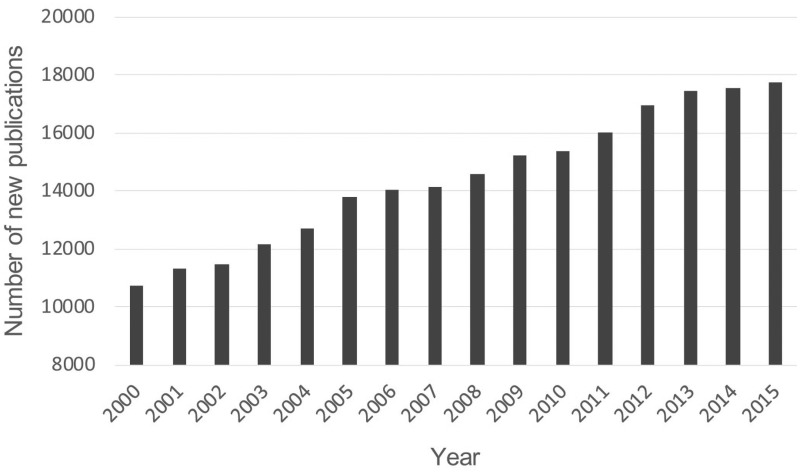
Number of new publications for immunosuppression from 2000 to 2015. The retrieval was performed using the keyword of ‘immunosuppression’ or ‘immunosuppressive’ in PubMed (https://www.ncbi.nlm.nih.gov/pubmed/).

All above information indicates that immunosuppression genes could be valuable drug targets or biomarkers for the immunotherapeutic treatment of different diseases. The number of new publications for immunosuppression is growing rapidly in recent years (Figure 1). However, the information of all previously identified immunosuppression genes is dispersed in thousands of publications. There is still no study emphasizing on the collection of immunosuppression genes. A comprehensive list of these genes is urgent for the study of human immunosuppression (2).

To address this need, we build the HisgAtlas database (http://biokb.ncpsb.org/HisgAtlas/) that collects 995 previously identified human immunosuppression genes using text mining and manual curation. HisgAtlas database 1.0 provides a user-friendly interface to search, browse, retrieve and download the information of human immunosuppression genes and their related drugs and diseases.

## Materials and methods

Our text mining is based on the immunosuppression-related abstracts from PubMed. Self-developed ontology-based bio-entity recognizer was used to perform bio-entity recognition and extraction from these abstracts for human immunosuppression gene candidates. Our recognition tool has the precision, recall and F-measure of 0.81, 0.88 and 0.85 against the CRAFT corpus for gene/protein recognition based on Protein Ontology (PR), which are comparable to current state-of-the-art biomedical annotation systems like BeCAS (13).

Three steps were taken to compile the list of human immunosuppression genes with their related diseases:

First, 120 176 PubMed abstracts and 180 063 sentences related to immunosuppression were collected with these keywords: ‘immunosuppression,’ ‘tumor immune escape,’ ‘inhibit T cell,’ ‘immunotolerance,’ ‘immune checkpoint,’ ‘immune deviation,’ ‘peripheral tolerance,’ ‘central tolerance,’ ‘allograft tolerance,’ ‘oral tolerance,’ ‘split tolerance,’ ‘self-tolerance,’ ‘natural tolerance,’ ‘acquired tolerance’ and their lexical variants.

Second, 3634 candidate human immunosuppression genes were recognized and extracted from these sentences based on PR (14) which co-occurred with the immunosuppression keywords at single-sentence level. That is to say, a gene occurs together with at least one of the immunosuppression keywords in a single sentence.

Third, three-round strict manual curation was performed on these candidates by our experts generating 995 high confidence human immunosuppression genes:

Round 1: All candidate immunosuppression genes and supporting evidence were checked by two experienced researchers independently.

Round 2: These selected genes and supporting evidence were submitted to the internal reviewer team, in which all immunosuppression genes were manually reviewed by three experts.

Round 3: All co-authors were asked to randomly check immunosuppression genes from our website to make sure that all immunosuppression genes stored in our database are of high confidence. Each co-author randomly checked 200 immunosuppression genes and 99.5% of them are correct on average.

Disease terms were also extracted from these abstracts based on Human Disease Ontology (DO) (15). Associations between immunosuppression genes/proteins and human diseases were identified based on single-sentence level co-occurrence. Furthermore, among these selected genes, those with the function of immune checkpoint were recognized through manual curation. A full list of immunosuppression-related membrane proteins is established based on the Gene Ontology Annotation (UniProt-GOA) Database (16) for the discovery of promising immune checkpoints as most of the immune checkpoints are membrane proteins.

Immunosuppression gene related drugs were extracted based on Drugbank (17). First, we extracted 410 drugs under ‘Immunoglobulins,’ ‘Immunoproteins,’ ‘Immunosuppressive Agents,’ ‘Antineoplastic and Immunomodulating Agents’ categories from Drugbank and then we mapped these drugs to immunosuppression genes using disease-drug relations extracted from the XML format of Drugbank. Finally, Manual validation was performed on these mappings to ensure data quality and 270 immunosuppression gene related drugs were obtained.

## Results

### Database search and navigation

We build the HisgAtlas database that collects 995 human immunosuppression genes as well as their related diseases. HisgAtlas provides a user-friendly web interface. It has two types of input for users: gene name for the gene query and disease name for the disease query.

**Figure 2.**
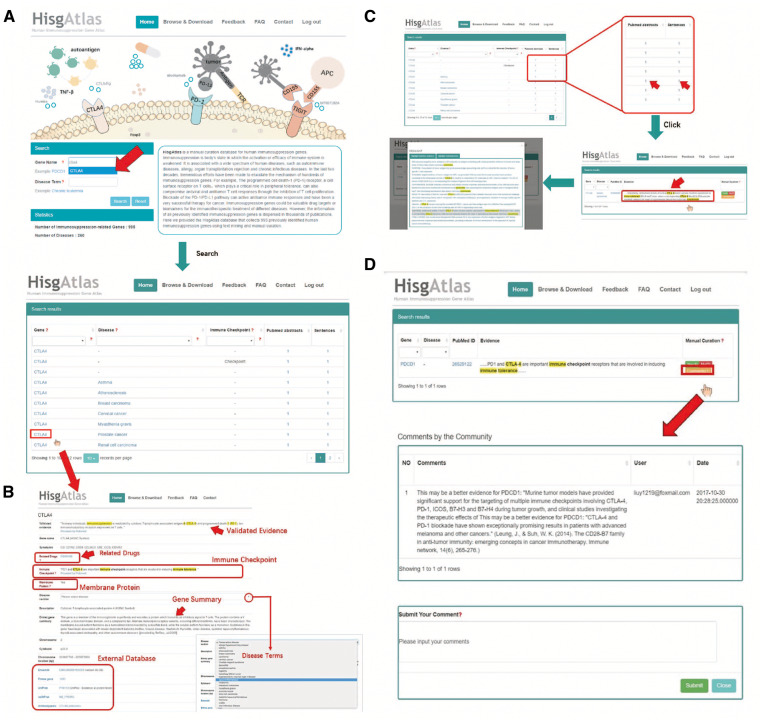
Outline of HisgAltas database website. (**A**) The users can submit a gene name in the ‘Gene name’ search box and find the information of this gene together with related diseases. (**B**) After clicking the gene name, users can see basic information about this gene and the cross references to external databases (i.e. Ensembl, Entrez Gene, UniProtKB). (**C**) After clicking the number of the evidence, the original evidence sentence will be displayed in which the key words are highlighted. Further clicking on individual evidence sentence of interest will lead to the view of whole abstract. (**D**) After login as registered users, one can simply click ‘Yes’ or ‘No’ button at the end of each evidence to confirm or disagree with the evidence. The user can also upload further detail information after clicking the ‘Comments’ button.

For example, CTLA4 is one of the most famous immunosuppression genes which can downregulate immune responses through competitively binding to CD80 or CD86 (18). Here, we searched our database with ‘CTLA4’ and the results revealed that CTLA4 might be involved in several diseases such as breast carcinoma, cervical cancer, etc. (Figure 2A, Supplementary Table S1). Further clicking on the gene name will lead to the gene interpretation page, including validated evidence on the top of this page and the gene info from Ensembl (19), the protein information from UniProtKB (20), the related drug information from Drugbank and the related disease information from DO. And the results also show that CTLA4 is a membrane protein and plays the role of immune checkpoint in cancer development (Figure 2B). After clicking the number of the evidence, the original evidence sentence will be displayed in which the keywords are highlighted. Further clicking on individual evidence sentence of interest will lead to the view of whole abstract (Figure 2C). To improve the confidence of gene-disease relations, we added a community curation function to supporting evidence with which users can easily provide their feedback by clicking the ‘Yes’ or ‘No’ button after login as registered users (Figure 2D). With this function, the users can help us to make sure that the evidence can support the corresponding gene-disease relation. The user can also upload further detail information after clicking the ‘Comments’ button.

All immunosuppression genes and their supporting literature evidence are available on the ‘Browse’ page and can also be downloaded. A well-described FAQ document was provided in our website (http://biokb.ncpsb.org/HisgAtlas/index.php/Home/Help/).

### Database implementation and design

HisgAtlas 1.0 currently contains 995 human immunosuppression genes and 260 related human diseases. All the information of these genes was loaded into a local MySQL database. Our website was developed using PHP and is currently running on a Windows server. The web service is available at http://biokb.ncpsb.org/HisgAtlas/.

Login is only needed if the user wants to participate in the community curation. All the other functions of HisgAtlas including data retrieval, browsing and downloading do not require any login or registration.

## Discussion

The inhibition of human immune system due to the presence of immunosuppression genes differs greatly from that due to the absence of immune response genes. Some of immunosuppression genes have been reported to be important immune checkpoints in cancer immunotherapy (2). However, the information of these immunosuppression genes occurs in thousands of papers (Figure 1) and construction of a systematic database for these genes would greatly deepen the understanding of immunopathogenesis, accelerate new findings of promising immune checkpoints and benefit the combinatorial immunotherapy of human disease. In this work, based on the literature mining and manual curation, we constructed the HisgAtlas 1.0 database containing 995 high confidence immunosuppression genes (Figure 2). This is the first database that emphasizes on the collection of immunosuppression genes.

**Figure 3.**
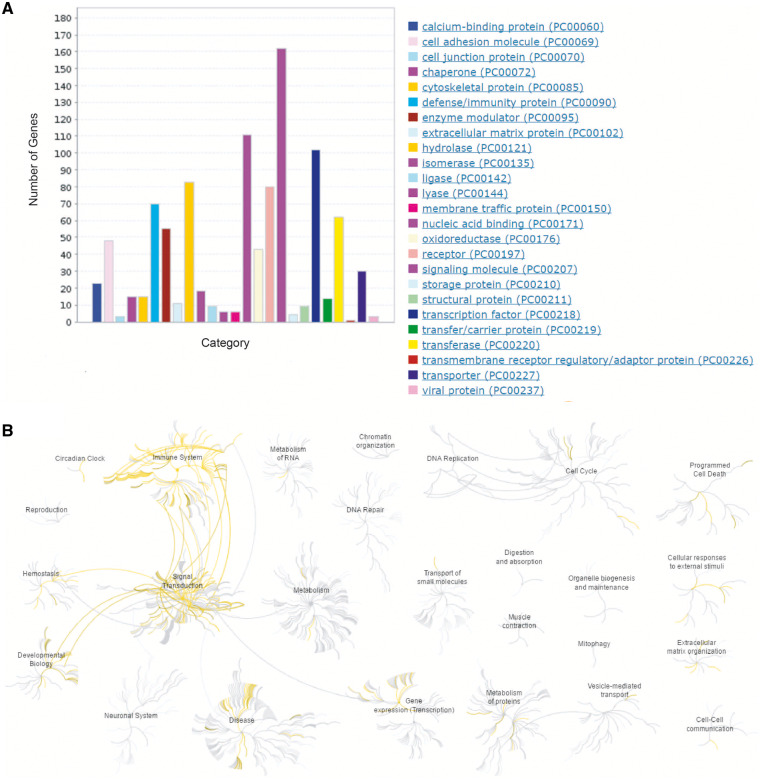
Bioinformatics analysis of the human immunosuppression-related genes. (**A**) Protein class analysis using PANTHER (http://pantherdb.org/). (**B**) Biological function and pathway analysis using Reactome (http://www.reactome.org/).

In addition, the comprehensive collection of HisgAtlas 1.0 database allows us to have an overview of human immunosuppression genes’ protein classes and their related biological pathways. Protein class analysis using PANTHER (21) shows these proteins are enriched in signaling molecule, nucleic acid binding, hydrolase, defense/immunity protein and transcription factors, receptor, etc. (Figure 3A, Supplementary Table S2). Biological function analysis using Reactome (22) indicates that human immunosuppression genes are actively involved in the immune system, signal transduction, gene expression, developmental biology, metabolism of proteins, hemostasis, etc. (Figure 3B, Supplementary Table S2). Pathway analysis using Reactome shows that these genes are actively involved in Signaling by GPCR (Reactom ID: R-HSA-372790), Interleukin-4 and 13 signaling (Reactom ID: R-HSA-6785807), Interferon gamma signaling (Reactom ID: R-HSA-877300), etc. (Supplementary Table S2).

However, several issues should be considered for HisgAtlas 1.0 database. First, some immunosuppression genes in the full texts of the literature are not shown in abstracts and might not be included in HisgAtlas, mostly due to that journals from major publishers are not freely available. Second, only one round of manual curation was performed on the immunosuppression gene related disease information, so we added the community curation function and hope it will help us to keep HisgAltas to be updated in the future.

In conclusion, we identified 995 immunosuppression genes and 260 related human diseases using text mining and manual curation. HisgAtlas 1.0 database is freely available to the scientific community. We believe it will be a valuable resource for scientific community to investigate the functions and mechanisms of human immunosuppression genes and their related diseases in the future.

## Author contributions

F.H. and D.L. conceived and conducted the research. Data collection, curation and analysis were performed by M.H., D.W., J. L. and Y.L. The manuscript was written and revised by Y.L., D.L., M.H., L.D. and S.G. Website was developed by L.D., M.H. and Y.L. All authors reviewed and approved the submitted manuscript.

## Funding

This work is funded by Program of Precision Medicine (2016YFC0901905), the Program of International S&T Cooperation (2014DFB30020), Chinese High Technology Research and Development (2015AA020108) and Beijing Nova Program (xx2014009).

## Supplementary data

Supplementary data are available at *Database* Online.

*Conflict of interest*. None declared.

## Supplementary Material

Supplementary Table 1

Supplementary Table 2
